# Supplementary material for: Active Compounds Derived from Fuzheng Huayu Formula Protect Hepatic Parenchymal Cells from Apoptosis Based on Network Pharmacology and Transcriptomic Analysis
Source: Molecules. 2019 Jan 18;24(2):338. doi: 10.3390/molecules24020338 (PMC6358846; doi:10.3390/molecules24020338)
Supplement: Supplementary file 1 [file molecules-24-00338-s001.zip › molecules-424611-final-SM/supplementary materials/Supplementary information Figs S1, S2, S3.pdf]

Article

Active compounds derived from fuzheng huayu formula protect hepatic parenchymal cells from apoptosis based on network pharmacology and transcriptomic analysis

Rong Wu[1]*, Shu Dong[1]*, Feifei Cai[1], Xiaole Chen[1], Mengdie Yang[1], Ping Liu[2], Shi-Bing Su[1]**

[1] Research Center for Traditional Chinese Medicine Complexity System, Shanghai University of Traditional Chinese Medicine, Shanghai, 201203, China

[2] E-institute, Shanghai University of Traditional Chinese Medicine, Shanghai, 201203, China

* co-first author

** Correspondence: shibingsu07@163.com; Tel.: +86 (0)21 5132 3013

Received: date; Accepted: date; Published: date

1.1.1 Supplementary Fig. S1a.

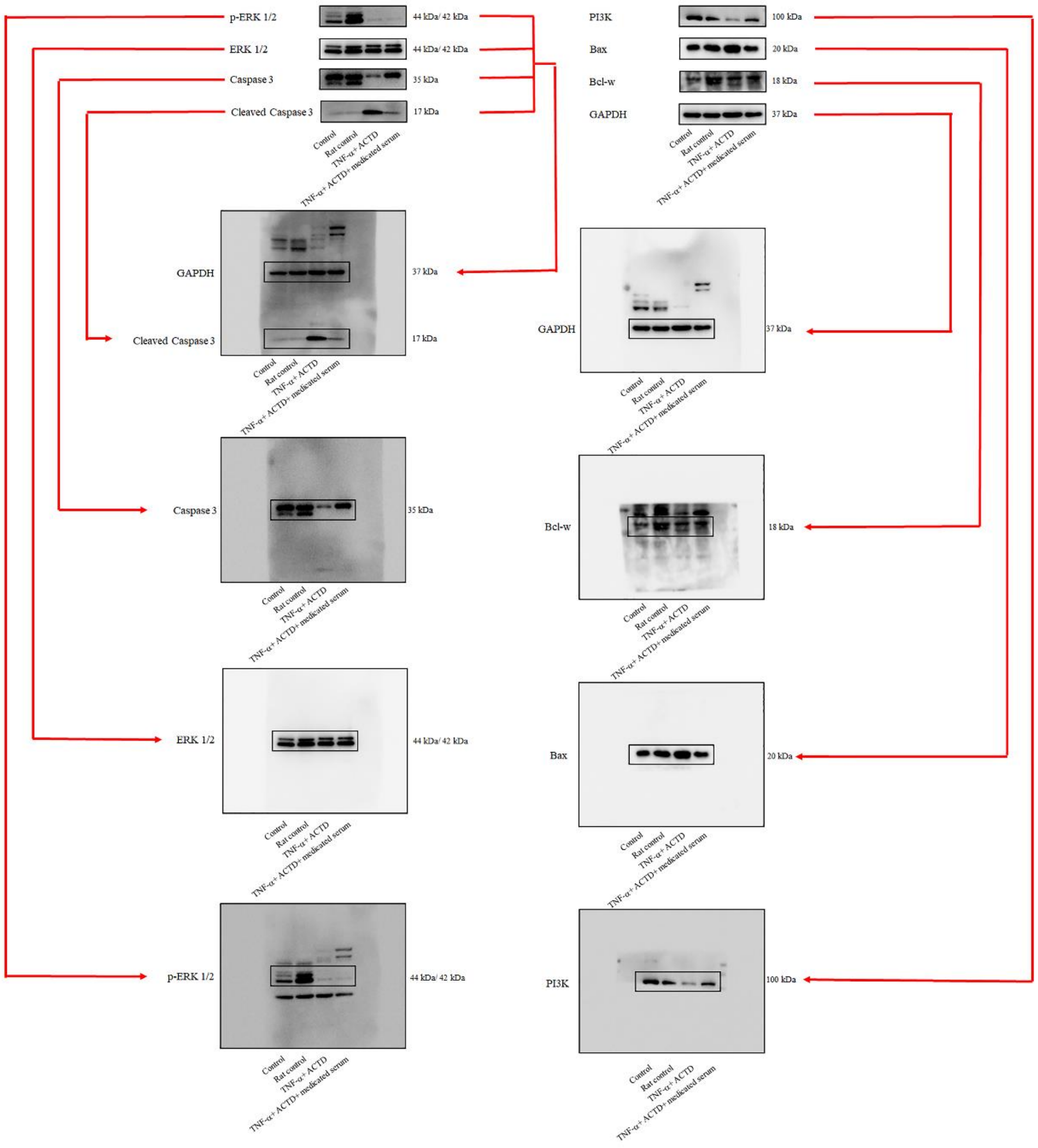

1.1.2 Supplementary Fig. S1b.

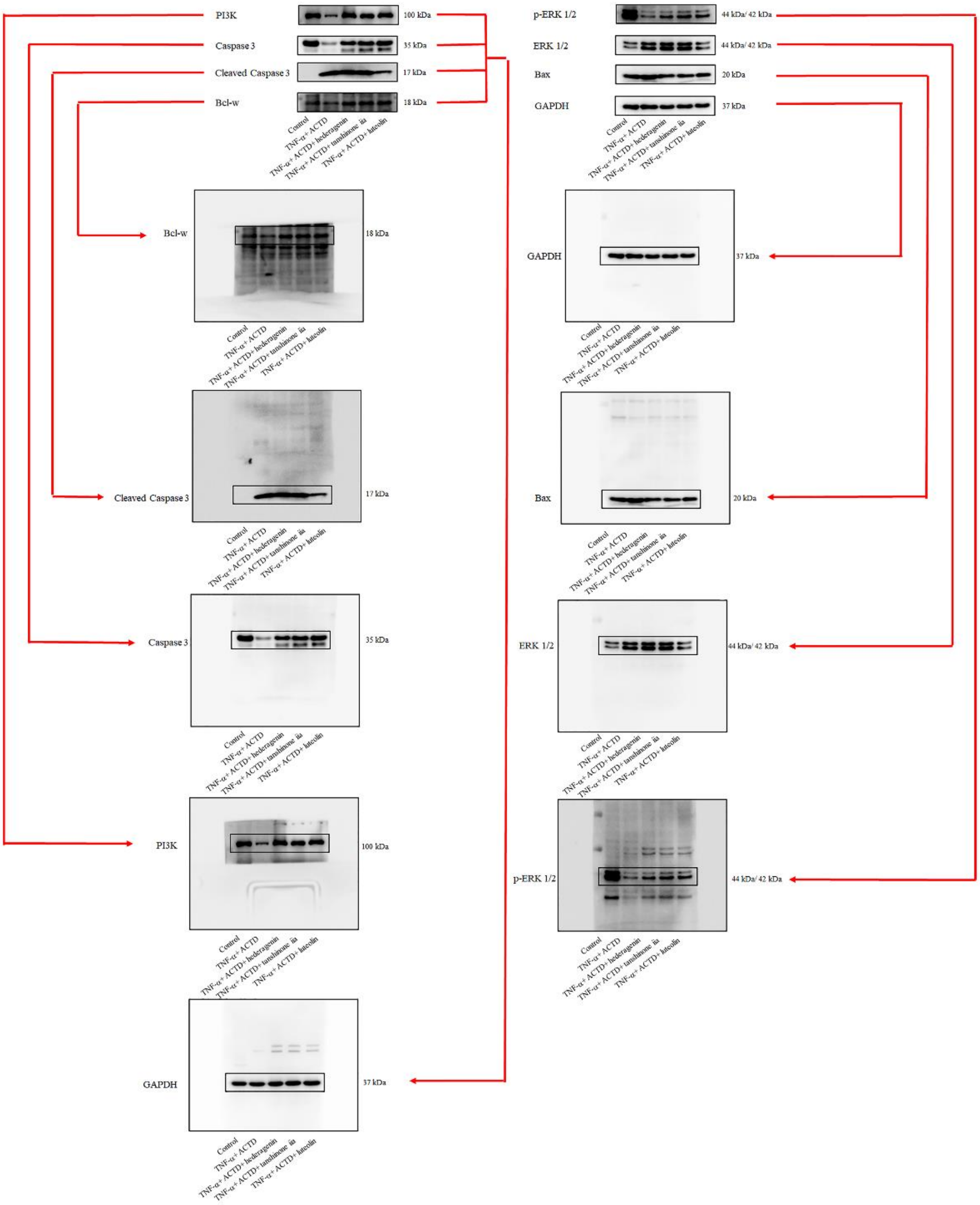

1.1.3 Supplementary Fig. S1c.

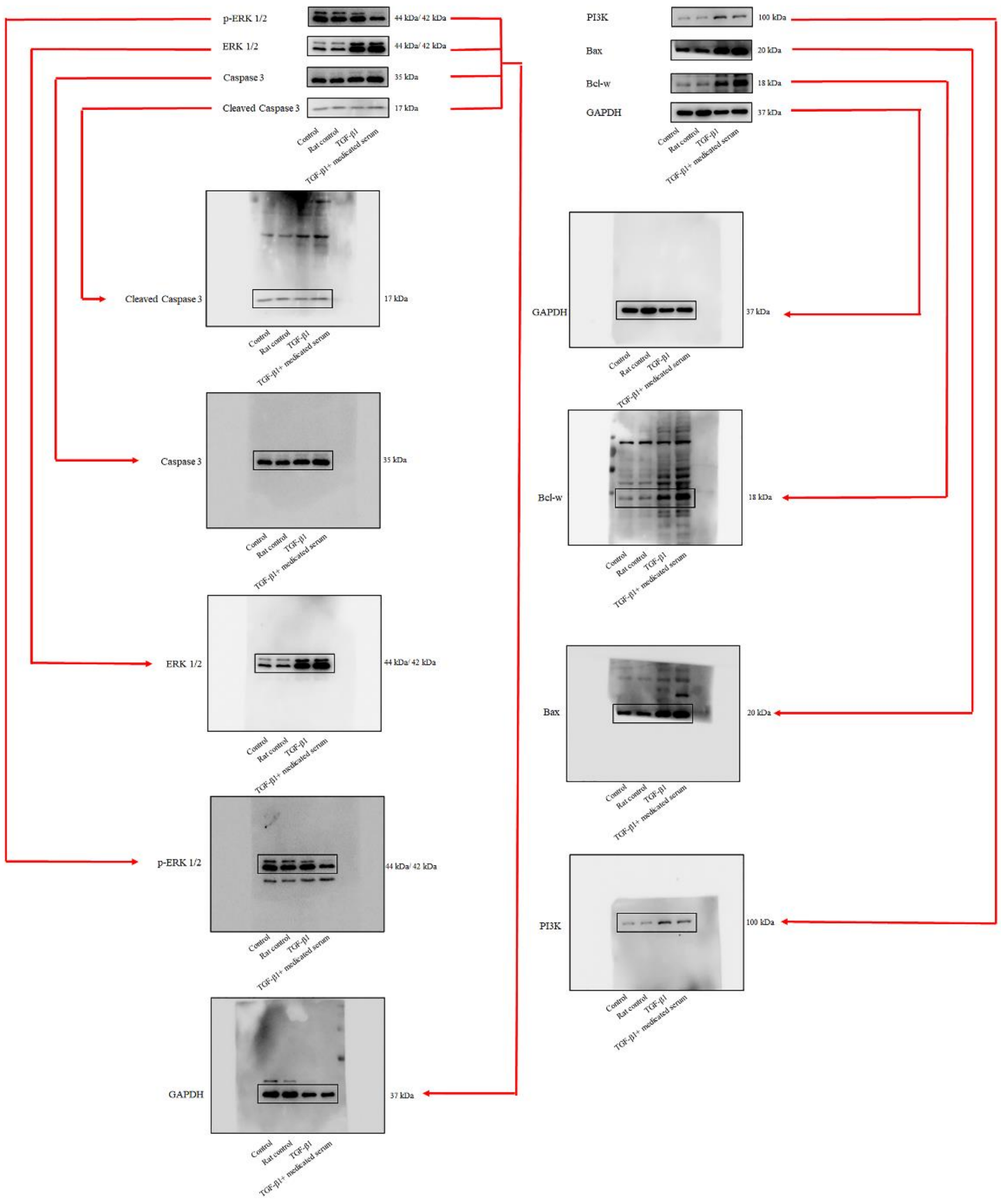

1.1.4 Supplementary Fig. S1d.

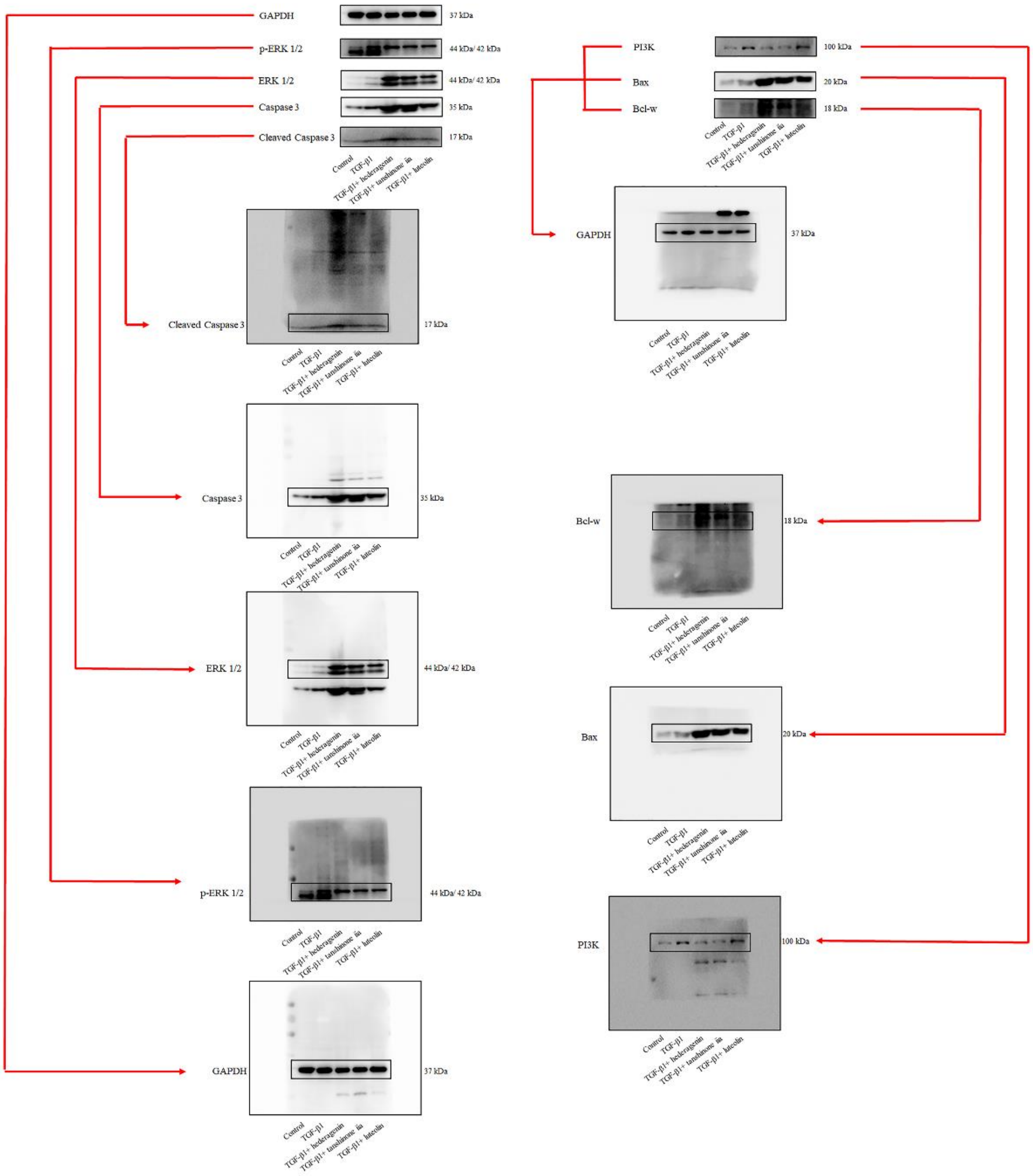

1.1.5 Supplementary Fig. S1e.

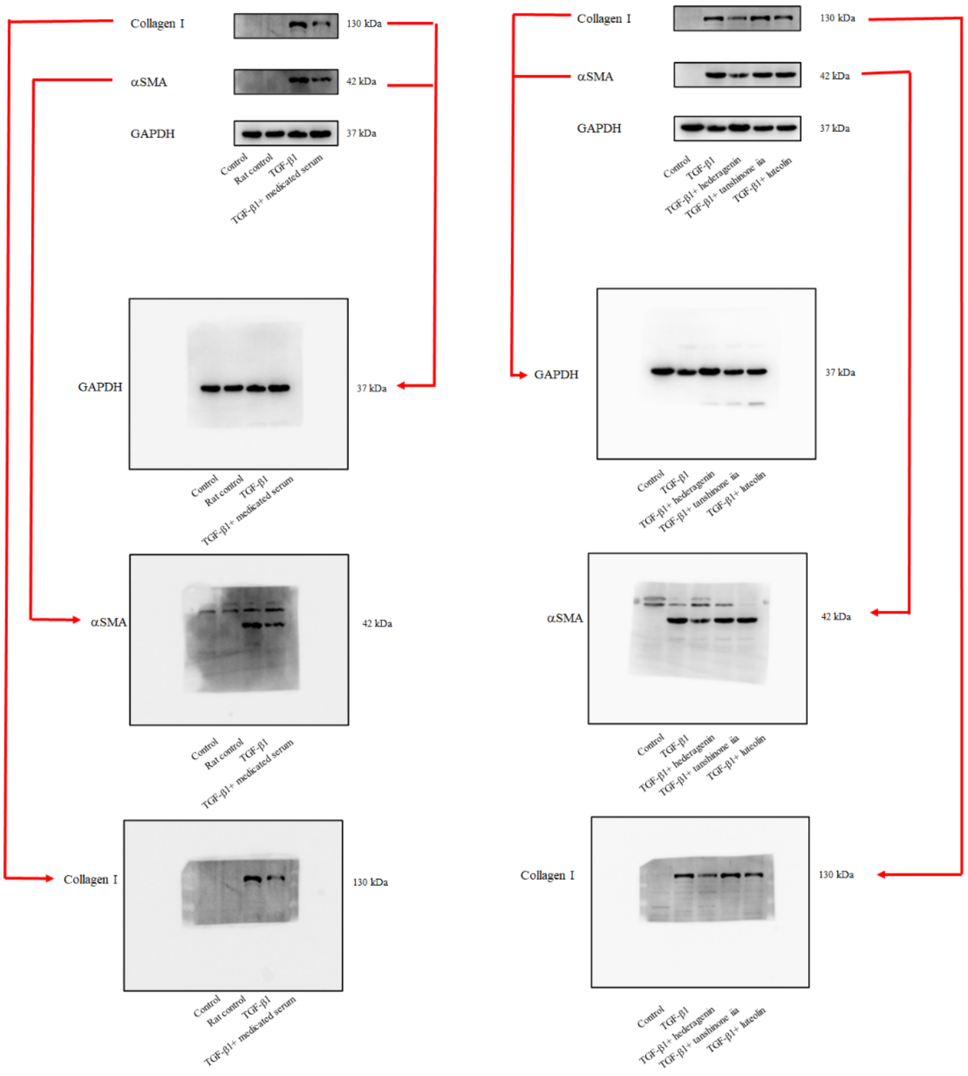

1.2 Supplementary Fig. S2.

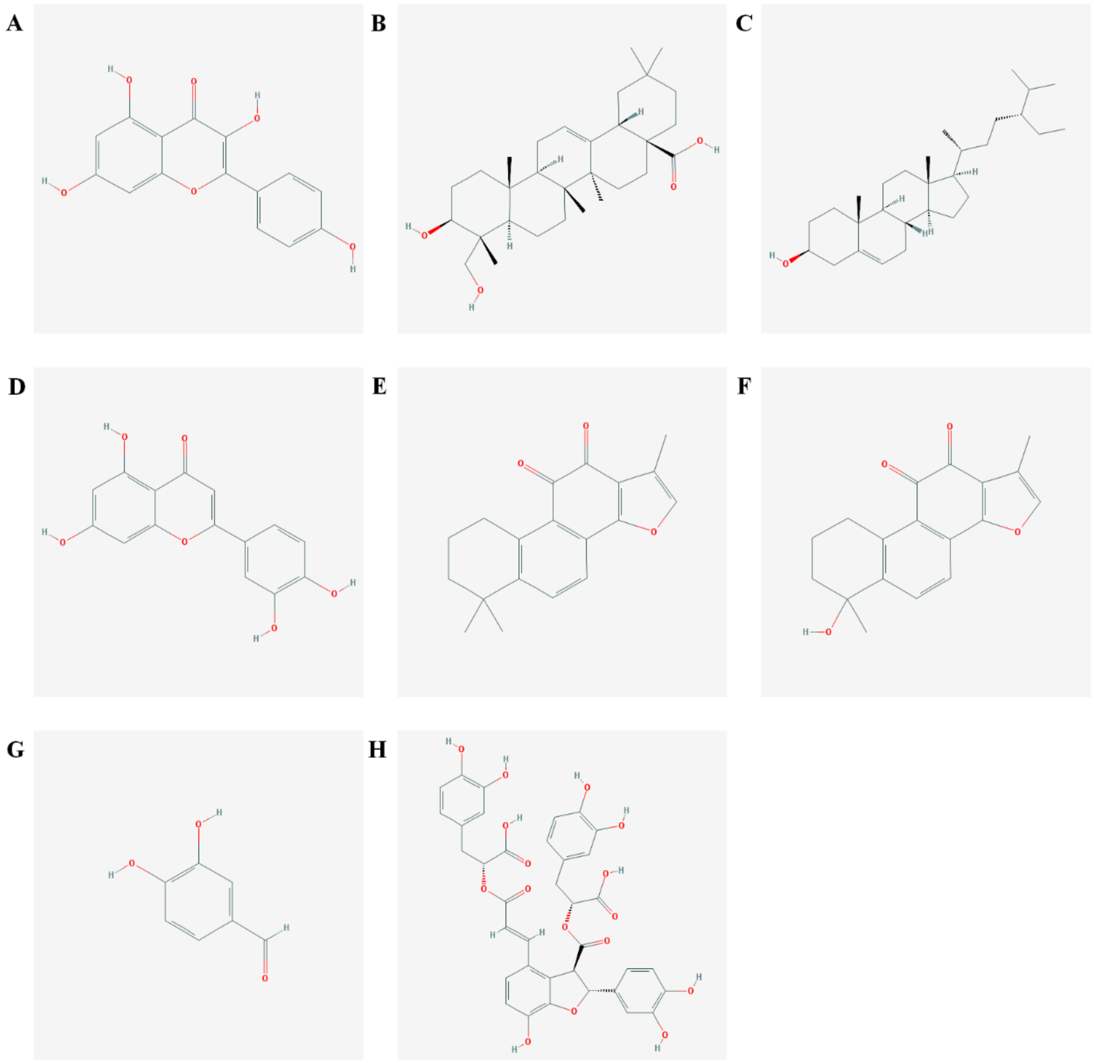

Supplementary Figure 2. Chemical structures of kaempferol, hederagenin, beta-sitosterol, luteolin and tanshinone iia. A, kaempferol; B, hederagenin; C, beta-sitosterol; D, luteolin; E, tanshinone iia; F, tanshinol B, G, protocatechualdehyde, H, salvianolic acid B.

1.3 Supplementary Fig. S3.

HPLC -MS MRM chromatogram of FZHY extracts and FZHY-medicated serum samples.

HPLC-MS conditions

Accela High performance liquid chromatography and LTQ Orbitrap XL were purchased from Thermo Fisher Scientific Company (USA); methanol (HPLC Grade) and formic acid (HPLC Grade) were purchased from Thermo Fisher Scientific Company (USA); reference standards were obtained from the Shanghai Institute of Materia Medica, Chinese Academy of Sciences.

FZHY extracts and FZHY-medicated serum sample were performed on high performance liquid chromatography (HPLC) Accela600 pump, LTQ Orbitrap XL

(Thermo Fisher Scientific Company, USA) using a SBAq column (4.6 × 250mm, 5 micron, Agilent Technologies, USA), Capillary Voltage 2500V–3000V, Tubeleu110V, Scan range100–1500, Sheath Gas 30 psi, and Aux Gas Flow 10 psi.

Method

The mobile phases comprised eluent A (0.1%formic acid) and eluent B (methanol). The gradient flow was as follows: 0~5minutes, 30% B; 5~40minutes, 30–90% B; 40~45 minutes, 90% to 100% B; 45~50minutes, 100% B. The analysis was performed at a flow rate of 1.0mL/min. The injection volumewas10μL.

Sample preparation

FZHY extracts were diluted into a concentration of 1mg/mL by 50% methanol-water solution, then the mix solution was ultrasonic extracted for 30 minutes at room temperature, filtered at 0.22 μm filter, stored at 4 ℃.

About 200 µl of the serum and 400 µl of acetonitrile were mixed and vortexed for 30 s. After centrifugation at 13,000 rpm for 10 min at 4 ℃. The concentrations of three compounds were 1mg/mL.

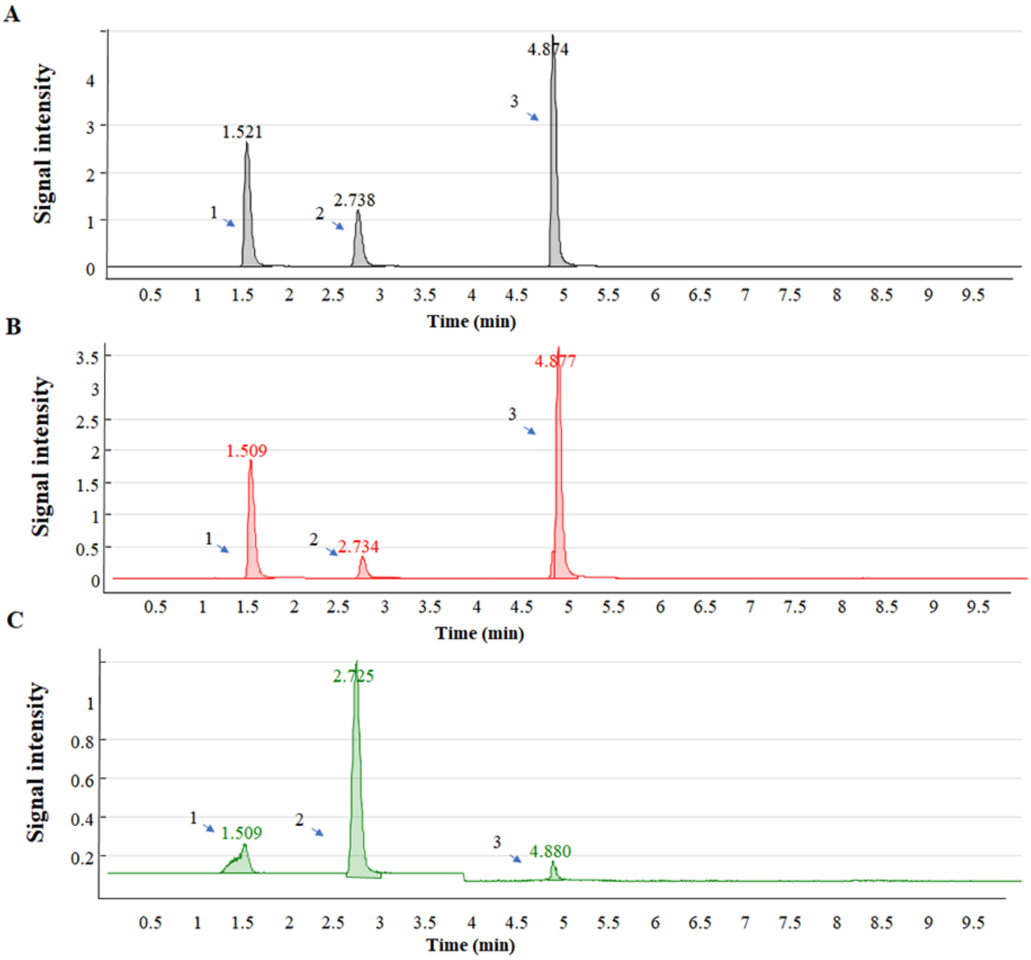

PEAK (A)	Area count
1	124139.08
2	63664.49
3	199006.21

PEAK (B)	Area count
1	83174.82
2	16305.97
3	145129.25

PEAK (C)	Area count
1	1452.29
2	6893.10
3	336.51

Supplementary Figure 3. Determination of tanshinol B, protocatechualdehyde and salvianolic acid B in FZHY extracts and FZHY-medicated serum sample by HPLC-MS MRM. A, morroniside, loganin and paeonol control; B, FZHY extracts; C, FZHY-medicated serum sample. 1, 2 and 3 represents tanshinol B, protocatechualdehyde and salvianolic acid B.
